# MiRNA-Mediated Knock-Down of Bcl-2 and Mcl-1 Increases Fludarabine-Sensitivity in CLL-CII Cells

**DOI:** 10.31557/APJCP.2021.22.7.2191

**Published:** 2021-07

**Authors:** Nooshin Ashofteh, Razieh Amini, Neda Molaee, Hadi Karami, Maryam Baazm

**Affiliations:** 1 *Department of Molecular Medicine and Biotechnology, Faculty of Medicine, Arak University of Medical Sciences, Arak, Iran. *; 2 *Molecular and Medicine Research Center, Arak University of Medical Sciences, Arak, Iran. *; 3 *Department of Anatomy, Faculty of Medicine, Arak University of Medical Sciences, Arak, Iran. *

**Keywords:** Bcl-2, CLL, Fludarabine, Mcl-1, MiRNA, 15a

## Abstract

**Background:**

Over-expression of anti-apoptotic proteins such as Bcl-2 and Mcl-1 is associated with resistance to chemotherapeutic agents such as fludarabine. Moreover, an inverse relationship between miRNA-15a levels with Bcl-2 and Mcl-1 expression has been observed in CLL patients. In this study, the effect of miRNA-15a on apoptosis and sensitivity of the CLL cells to fludarabine was investigated.

**Methods:**

After treatments, the Mcl-1 and Bcl-2 expression levels were quantified by RT-qPCR. Trypan blue assay was used to explore the effects of miRNA-15a and fludarabine on cell proliferation. The cytotoxicity was measured using MTT assay and combination index analysis. Cell death was determined using cell death detection ELISA assay and caspase-3 activity assay Kits.

**Results:**

Results showed that miRNA-15a clearly decreased the mRNA levels of Bcl-2 and Mcl-1 in a time dependent manner, which led to CLL-II cell proliferation inhibition and enhancement of apoptosis (p < 0.05, relative to control). Transfection of the miRNA-15a synergistically reduced the cell survival rate and lowered the IC_50_ value of fludarabine. Furthermore, miRNA-15a significantly enhanced the apoptotic effect of fludarabine.

**Conclusions:**

Our data propose that suppression of Bcl-2 and Mcl-1 by miRNA-15a can effectively inhibit the cell proliferation and sensitize CLL cells to fludarabine. Therefore, miRNA-15a can be considered as a potential therapeutic target in CLL resistant patients.

## Introduction

Chronic lymphocytic leukemia (CLL) is a haematological malignancy of B lymphocytes and the most prevalent type of leukemia in adults (Danilov, 2013; Khan and Siddiqi, 2018). Therapy available for CLL includes chemotherapy with agents such as fludarabine, along with immunotherapy including alemtuzumab (Khan and Siddiqi, 2018). Fludarabine (9-b-D-arabinofuranosyl-2-fluoroadenine), a purine nucleoside analog, is one of the most active single therapeutic agents in the treatment of CLL. Due to their potential to kill non-dividing cells, fludarabine produces good response and often is used as first and second-line treatment in CLL patients (Ferracin et al., 2010; Moussay et al., 2010). However, virtually all of patients receiving fludarabine is either initially resistant or becomes resistant to this agent in the years. The exact molecular mechanisms underlying this resistance are only poorly described (Ferracin et al., 2010; Nana-Sinkam and Croce, 2010).

Apoptosis is triggered by the two intrinsic and extrinsic pathways. The intrinsic pathway of apoptosis is controlled by the members of the Bcl-2 family proteins including the pro-survival members and the pro-apoptotic members (Shahverdi et al., 2020a; Shahverdi et al., 2020b). Up-regulation of prosurvival Bcl-2 family members such as Bcl-2 and Mcl-1 is associated with resistance to treatment with fludarabine, chlorambucil and rituximab, as well as shorter overall survival in CLL patients. Accordingly, many literatures have indicated that targeted down-regulation of prosurvival Bcl-2 proteins is an pertinent strategy for apoptosis-based therapeutics in CLL (Billard, 2012; Fegan and Pepper, 2013).

MicroRNAs (miRNAs) are short single-stranded RNAs of about 19-24 nucleotides that bind directly to the 3’ untranslated region (3’-UTR) of the specific target mRNA, leading to inhibition of target expression or mRNA degradation (Nazarian et al., 2019; Alamdari-Palangi et al., 2020a). MiRNAs have recently been identified as one of the new biomarkers in several types of cancer (Alamdari-Palangi et al., 2020b). In addition, miRNAs are involved in various processes such as lymphocyte development and differentiation, indicating the important role of miRNAs in CLL (Calin et al., 2008). Chromosomal abnormalities are seen in more than 80% of CLL cases. These aberrations include the 13q, 11q and 17p deletions, which cause various outcomes in patients (Hallek, 2019). Previous studies have shown that deletion of 13q14 is observed in more than 50% of CLL cases. The results of other reports have clarified that the deleted region includes a deletion in the miRNA-15a gene (Lia et al., 2012). MiRNA-15a acts as a tumor suppressor gene and is involved in the regulation of cell growth and apoptosis through inhibiting several targets such as Bcl-2, cyclin D1, cyclin D3 and cyclin-dependent kinase 6 (CDK6) (Liu et al., 2008). The results of microarray experiments also show an inverse relationship between high or low miRNA-15a levels with the expression of Mcl-1 gene in CLL patients (Calin et al., 2008). However, the role of miRNA-15a in pathogenesis and chemoresistance of CLL has not been fully elucidated.

We hypothesized that down-regulation of miRNA-15a could enhance the chemoresistance of the CLL cells via the blockage Bcl-2 and Mcl-1 expression. Therefore, we investigated the effect of miRNA-15a on apoptosis and sensitivity of the CLL cells to fludarabine.

## Materials and Methods

*Cell culture*

The human CLL-CII cell line (Pasteur Institute, Tehran, Iran) was cultured in RPMI-1640 medium (Sigma-Aldrich, St. Louis, MO, USA) supplemented with 15% heat-inactivated FBS (Sigma-Aldrich), 1% sodium pyruvate, 2 mM of glutamine, 100 U/ml penicillin-streptomycin (Sigma-Aldrich) and incubated at 37°C in 5% CO_2_. The cancer cells were cultivated at an initial density of 1×10^5^ cells/ml. Cells were subcultured at 80% confluence and used in exponentially growing phase in all experiments.

*Cell transfection*

The miRNA-15a mimics with the sense strand sequence 5’-UAG CAG CAC AUA AUG GUU UGU G-3’ and the negative control (NC) miRNA sense strand sequence 5’-ACU ACU GAG UGA CAG UAG A-3’ were purchased from Dharmacon (Lafayette, CO, USA) and used in transient transfection of CLL cell line. The cells were transfected with miRNA-15a mimics or NC miRNA at a final concentration of 50 nM, using Lipofectamine™2000 transfection reagent (Invitrogen, Carlsbad, CA, USA) and Opti-MEM I reduced serum medium (Invitrogen) according to the manufacturer’s instructions. Optimal concentrations of miRNA and Lipofectamine for transfection were determined empirically.

*MTT assay*

The effect of miRNA-15a on the sensitivity of CLL-CII cells to fludarabine (Sigma- Aldrich) was determined using 3-(4, 5-Dimethylthiazol-2-yl)-2, 5 Diphenyltetrazolium Bromide (MTT) assay. The experiment was subdivided into eight groups: miRNA-15a mimics, NC miRNA, fludarabine, miRNA-15a mimics and fludarabine, NC miRNA and fludarabine, miRNA blank control, fludarabine blank control and combination blank control. In brief, cells were cultured at a density of 5×104 cells per well in 96-well plates and then transfected with miRNAs. Six hours after transfection, fludarabine with different concentrations (0, 0.05, 0.1, 0.2, 0.4, 0.8, 1.6 and 3.2 µM) were added. 

Twenty-four and forty-eight hours after transfection, the cell toxicity was determined using the MTT cell assay kit (Roche Diagnostics GmbH, Mannheim, Germany) according to the manufacturer’s protocol. Absorbance (A) at 490 nm (with a reference wavelength of 650 nm) was read using a microplate spectrophotometer (Awareness Technology, Palm City, FL, USA). Half maximal inhibitory concentration (IC_50_) value was calculated using GraphPad Prism 6.01 software (GraphPad Software Inc., San Diego, CA, USA). In subsequent experiments, the IC_50_ dose of fludarabine was used. 

*Drug combination study*

The combination index (CI) analysis based on the Chou-Talalay method was performed to explore the interaction between miRNA-15a and fludarabine (Amri et al., 2019a; Amri et al., 2019b). The results obtained from the MTT assay was converted to Fraction affected (Fa; range 0-1; where Fa = 1 represents 0% cell survival and Fa = 0 represents 100% cell survival) and analyzed with the CompuSyn software (ComboSyn Inc., Paramus, NJ, USA). A CI of >1, =1 or < 1, indicates antagonistic, additive and synergistic effects, respectively.

*QRT-PCR*

Following treatments, total RNA was extracted from CLL-II cells using TRIzol reagent (Invitrogen) as described by the manufacturer’s protocol. Reverse transcription was performed from 1 μg of total RNA using AMV reverse transcriptase and random primers (Promega, Madison, WI, USA) according to the manufacturer’s instructions. The cDNAs were amplified with SYBR Premix Ex Taq (Takara Bio, Otsu, Shiga, Japan) and the LightCycler 96 System (Roche Diagnostics GmbH, Mannhein, Germany). QRT-PCR was performed in a final volume of 20 µl containing 10 µl of SYBR green reagent, 1 µl of cDNA, and 500 nM of each of the primers. The PCR reactions were performed under the blow procedure (95°C 5 min; 95°C 10 sec, 60°C 30 sec, 72°C 30 sec, 40 cycles). The primers were: forward, 5’-TCC CTG GAG AAG AGC TAC G-3’, reverse, 5’-GTA GTT TCG TGG ATG CCA CA-3’, for β–actin, forward, 5’-ATC GCC CTG TGG ATG ACT GAG T-3’, reverse, 5’-GCC AGG AGA AAT CAA ACA GAG GC-3’, for Bcl-2, and forward, 5’-TAA GGA CAA AAC GGG ACT GG-3’, and reverse, 5’-ACC AGC TCC TAC TCC AGC AA-3’, for Mcl-1. The relative mRNA level was determined using the 2-(∆∆Ct) method (Pirayesh Islamian et al., 2016; Mohammadi et al., 2017) and the fold change in gene expression was normalized to a reference gene (β-actin).

*Cell proliferation assay*

The antiproliferative effect of miRNA-15a and fludarabine was measured by trypan blue assay. Cells (1×10^5^ cells per well) were treated with miRNA-15a and fludarabine in 6-well cell culture plates and then incubated for 1-5 days. At different time points after treatment, the cells were harvested and the cell suspensions were stained with equal volume of 0.4% trypan blue (Merck KGaA, Darmstadt, Germany). After 2 min, the number of viable cells (N) was counted using a hemocytometer under an inverted microscope (Nikon Instrument Inc., Melville, NY, USA). The percentage of viable cells in test groups was determined by the formula as follows: Cell viability (%)= (N test /N control) ×100. The percentage of viable cells in blank control group in each time was considered as 100%.

*Apoptosis ELISA assay*

The CLL-CII cells were seeded at a density of 1×10^5^ cells per well in 6-well plates and then treated with fludarabine, miRNA-15a, NC miRNA, and their combination, as described previously. At 24 and 48 h after transfection, cells were harvested and apoptosis was determined using an ELISA cell death detection kit (Roche Diagnostics GmbH) according to the manufacturer’s recommendation. This assay measures the amount of mono- and oligonucleosomes produced during apoptosis. Briefly, 20 μl of the supernatants were transferred into each well of a streptlized-coated plate and incubated with a mixture of anti-histone-biotin and anti-DNA-peroxidase. Following color development with ABTS solution, the reactions were stopped and absorbances of the samples were quantified with an ELISA plate reader at 405 nm (reference wavelength 540 nm). Data were presented as the fold increase in apoptosis as compared with blank group.

*Caspase-3 activity assay*

The in vitro caspase-3 activity was determined using a colorimetric caspase activity assay Kit (Abnova Corporation, Taipei, Taiwan). The treated cells were lysed in lysis buffer and the cell suspensions were centrifuged in 10,000 g for 1 min. Then, 50 µg of supernatant and 50 µl of 2X reaction buffer was transferred to a fresh tube. Then, the caspase-3 (DEVD-pNA) colorimetric substrate with a final concentration of 200 µM was added into each sample and incubated for 2 h at 37°C. The absorbance was measured at 405 nm.

*Statistical analysis*

Quantitative data in this study are presented as mean ± standard deviation (SD). Statistical significance of differences between the means of the two groups was explored by the analysis of variance (ANOVA) and Bonferroni’s test using GraphPad Prism software. Value of P less than or equal to 0.05 was considered significant. 

## Results

*MiRNA-15a enhanced the sensitivity of the leukemia cells to fludarabine*

**Figure 1:**
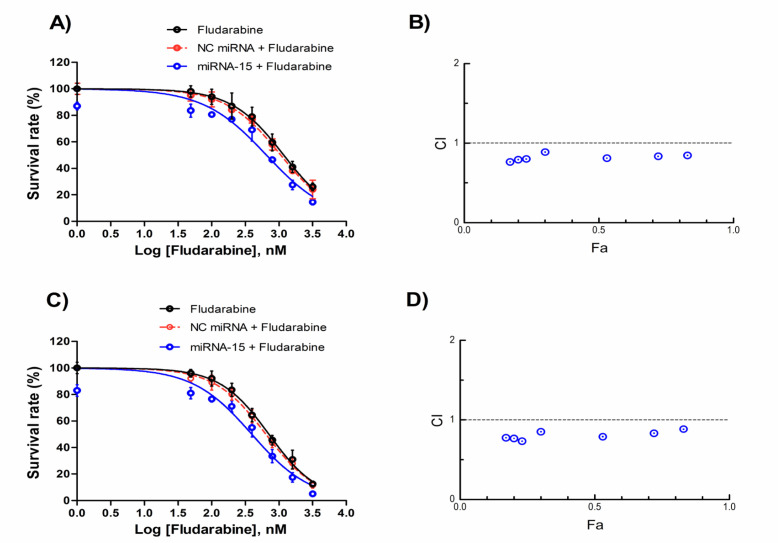
The Effect of miRNA-15a on Sensitivity of the CLL-CII Cells to Fludarabine. Cells were treated with miRNA-15a (50 nM) and different concentrations of fludarabine for 24 h (A and B) and 48 h (C and D). Next, the cell survival rate was determined using MTT assay. Cell survival curves were plotted by Prism software. The data are expressed as mean ± SD (n=3). Results from three independent experiments were used to plot the combination index (CI) versus fractional effect (Fa) using the Chou and Talalay method and CalcuSyn software. Dashed lines represent CI=1.

To analyze whether mRNA-15a could enhance the sensitivity of the CLL-CII leukemia cells to fludarabine, a combination treatment of miRNA-15a and fludarabine was investigated. The results of MTT assay demonstrated that monotreatment with fludarabine reduced the cell survival in a dose and time dependent manner. As shown in Figure 1A and 1C, 24 and 48 h after transfection of miRNA-15a, the cell survival rate was significantly decreased to 86.50% and 81.41% respectively, compared with the blank control group (p<0.05). Moreover, miRNA-15a in combination with fludarabine further reduced the cell survival rate relative to miRNA-15a or fludarabine alone (p<0.05). Surprisingly, miRNA-15a caused a significant reduction in the IC_50_ values of fludarabine from 1.17 µM to 0.65 µM and 0.70 µM to 0.39 µM after 24 and 48 h, respectively (Table 1). Meanwhile, NC miRNA had an insignificant effect on cell survival and chemosensitivity of the CLL cells relative to the blank control or fludarabine treated cells (p>0.05; Figure 1 and Table 1).

*MiRNA-15a acts synergistically with fludarabine to decrease the cell survival of CLL cells*

To further explore whether the decrease in cell survival was the synergistic effect of the miRNA-15a and fludarabine, combination index analysis was performed on MTT assay results using non-constant method of Chou and Talalay. The CI–Fa curves showed a synergism (CI<1) in CLL-CII cells when miRNA-15a (50 nM) combined with fludarabine (0.05-3.2 µM) (Figure 1B and 1D). Our data demonstrated that the best mean CI value of 24 h of treatment (CI=0.76) was obtained at 0.05 µM fludarabine with Fa level of 0.17. Moreover, at 0.2 µM fludarabine with Fa level of 0.23 the best mean CI value for 48 h (CI=0.73) was observed (Table 2).

*MiRNA-15a down-regulated mRNA expression levels of Mcl-1 and Bcl-2 in CLL-CII cells*

**Figure 2:**
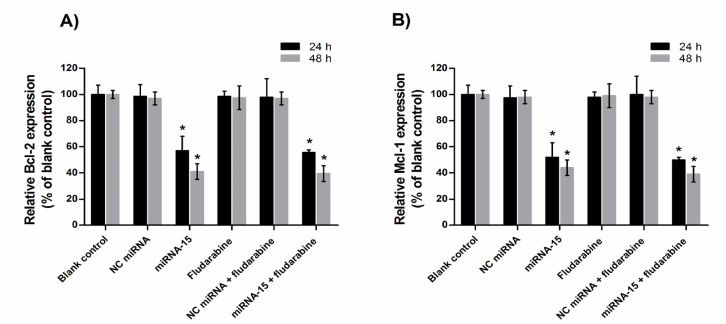
Gene Expression Analysis in CLL-CII Cells Treated with miRNA-15a and Fludarabine. The cells were treated with miRNA-15a, fludarabine and combination of them for 24 and 48 h. Relative Bcl-2 (A) and Mcl-1(B) mRNA expression was measured using RT-qPCR and 2^ - (∆∆Ct)^ method. The results are presented as mean±SD of the results of three experiments. *p<0.05 versus corresponding blank control or NC miRNA transfected cells

Firstly, we explored the effect of miRNA-15a and fludarabine on Mcl-1and Bcl-2 expression in CLL cells by qRT-PCR. Relative gene expression was calculated compared with the blank control group, which was considered as 100%. Results of qRT-PCR demonstrated that 24 and 48 h transfection of miRNA-15a markedly decreased the expression levels of Bcl-2 and Mcl-1 mRNA relative to the blank control group (p<0.05; Figure 2). No significant effects on mRNA levels of Mcl-1and Bcl-2 were observed in solvent control, NC miRNA or fludarabine groups (p>0.05; compared with blank control). In addition, in cells treated with combination of fludarabine and miRNA-15a, the expression of Bcl-2 and Mcl-1 mRNA was not different from miRNA-15a-treated cells (p>0.05). 

*MiRNA-15a enhanced the effect of fludarabine on cell proliferation*

**Figure 3:**
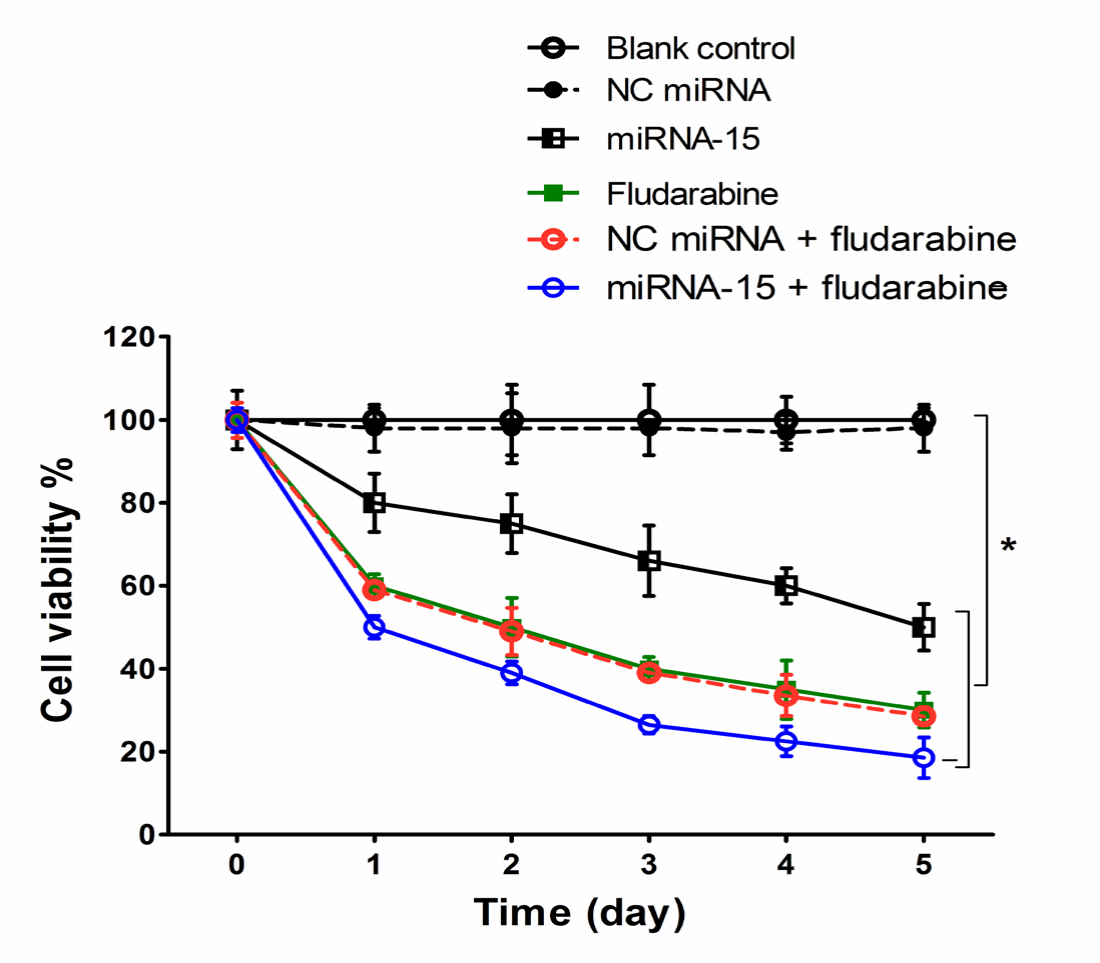
Proliferation Curve of CLL-CII Cells Treated with miRNA-15a and Fludarabine. Cell proliferation was measured using trypan blue exclusion assay over a period of 5 days. Results are expressed as mean ± SD (n=3). *p< 0.05 versus blank control or NC miRNA

As up-regulation of Mcl-1 and Bcl-2 is associated with proliferation and survival of the tumor cells; we therefore sought to assess whether miRNA-15a could inhibited the proliferation of CLL cells. Results showed that after 24 h treatment of the cells with miRNA-15a and fludarabine, the percentage of cell viability reduced to 81.63% and 62.28% respectively, relative to the blank control group (p<0.05; Figure 3). Reduction of cell viability continued in the next days and reached to 49.37% and 32.37% on day 5 for miRNA-15a and fludarabine, respectively. In combination therapy with miRNA-15a and fludarabine, a greater reduction in cell viability was observed than monotherapy during this 5-day period (p<0.05). No significant alteration in cell viability was observed between the blank control group and NC miRNA transfected cells.

*MiRNA-15a sensitized chronic lymphocytic leukemia cells to apoptosis induced by fludarabine*

**Figure 4:**
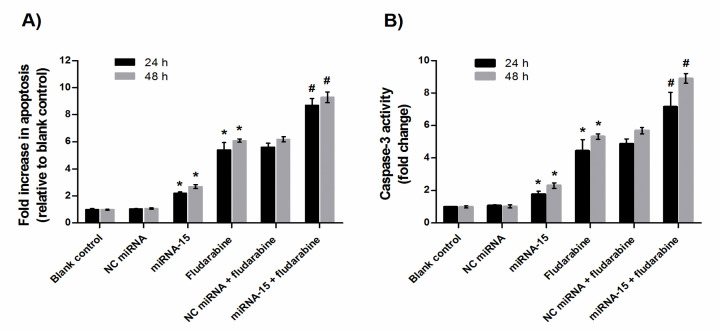
Combination Effects of miRNA-15a and Fludarabine on Apoptosis of CLL Cells. Cells were treated with miRNA-15a (50 nM), negative control (NC) miRNA (50 nM) and fludarabine (IC50 doses of 24 and 48 h). Next, the apoptosis was quantified using ELISA apoptosis assay (A). Caspase-3 activity of CLL cells was detected according to caspase-3 activity assay Kit (B). The data are presented mean ± SD (n=3) of three independent experiments. *p<0.05 compared with control; #p<0.05 versus miRNA-15a or fludarabine monotreatment

To confirm whether the sensitizing effect of the miRNA-15a was related to the increase in the extent of apoptosis, the effects of miRNA-15a, fludarabine and their combination on apoptosis were evaluated using an ELISA-based cell death detection assay. As shown in Figure 4A, 24 h after transfection of miRNA-15a alone, apoptosis increased by 2.20 fold, whereas fludarabine treatment alone led to 5.45 fold increase in apoptosis (p<0.05, compared to the blank control). Moreover, the combination therapy further increased apoptosis to 8.74 fold (p<0.05, compared with single therapy). After 48 h of treatment of CLL-CII cells to miRNA-15a or fludarabine alone, apoptosis increased by 2.70 and 6.10 fold, respectively, compared to the blank control (p<0.05). Also, combination treatment for 48 h enhanced the extent of apoptosis to 9.30 fold after (Figure 4A; p<0.05, relative to the blank control or monotreatment). On the other hand, treatment with NC miRNA alone or in combination with fludarabine displayed no significant effect on apoptosis. Therefore, these data suggest that the chemosensitization effect of miRNA-15a in CLL cells is partially attributed to the induction of apoptosis.

*MiRNA-15a enhanced the effect of fludarabine on caspase-3 activity in CLL cells*

To explore the mechanism by which apoptosis triggered in the tumor cells, changes in the activation of the caspases-3 were measured. Figure 4B shows the changes in caspases-3 activity in the CLL cells treated with the miRNA-15a, fludarabine and their combination for 24 h that indicate the caspase-3 activity was increased by 1.77, 4.36, and 7.18 times, respectively, compared with blank control cells (p<0.05). As indicated in Figure 4B, miRNA-15a alone and in combination with fludarabine activated caspase-3 activity in a time dependent manner. NC miRNA did not cause a significant change in caspase-3 activity compared to the control group (p>0.05).

**Table 1:** IC_50_ Values of Fludarabine, Alone and in Combination with miRNAs, in CLL-CII Cells

Treatment	IC_50_ (µM)	
	24 h	48 h
fludarabine	1.17 ± 1.35	0.70 ± 1.30
NC miRNA and fludarabine	1.06 ± 1.28^#^	0.64 ± 0.80^#^
miRNA-15a and fludarabine	0.65 ± 2.31*	0.39 ± 1.13*

IC_50 _values were calculated by sigmoidal dose-response model using Prism software. Data presented as the mean ± SD of three experiments. *p<0.05 relative to the corresponding fludarabine; #p>0.05 relative to the corresponding fludarabine.

**Table 2:** CI Analysis of miRNA-15a and Fludarabine Combination in CLL-CII Cells

Fludarabine concentration (µM)	24 h			48 h		
	Fa	CI	Combined effect	Fa	CI	Combined effect
0.05	0.17	0.76	S	0.18	0.77	S
0.1	0.2	0.79	S	0.21	0.76	S
0.2	0.23	0.8	S	0.23	0.73	S
0.4	0.3	0.88	S	0.33	0.85	S
0.8	0.53	0.81	S	0.54	0.78	S
1.6	0.72	0.83	S	0.7	0.8	S
3.2	0.83	0.84	S	81	0.88	S

The combination index (CI) values were calculated using Chou-Talalay combination index method and CompuSyn software. Antagonistic, additive and synergistic (S) effects are defined by CI value >1, =1 and >1, respectively.

## Discussion

Fludarabine is one of the most effective single agents often used as first-line therapy in CLL patients (Zhou and Wang, 2013). Although fludarabine has shown considerable activity against CLL, however, virtually all CLL patients do not respond or becomes resistant to this agent in the years. It has been reported that genetic factors such as p53 dysfunction, deriving from 17p13 deletion, 11q22.3 deletion and immunoglobulin (Ig) V (H) unmutated status have been associated with fludarabine resistance (Ferracin et al., 2010). Moreover, deregulation in apoptosis and expression of miRNAs is also related to this process. However, the exact molecular mechanisms are not fully understood (Fegan and Pepper, 2013). 

The results of our study showed that suppression of Mcl-1 and Bcl-2 expression by miRNA-15a was associated with inhibition of cell growth and synergistically increased the sensitivity of CLL cells to fludarabine. In accordance with the results of our study, Robertson et al. (1996) showed that high expression levels of Bcl-2 is correlated with shorter overall survival and resistance to ﬂudarabine in CLL patients. Other reports indicated that high levels of Mcl-1 in the leukemic lymphocytes were associated with resistance to rituximab, chlorambucil and ﬂudarabine both in vitro and in vivo (Billard, 2012; Fegan and Pepper, 2013). In addition, Hussain et al., (2007) showed that targeted down-regulation of Mcl-1 is enough to trigger apoptosis and enhance rituximab-mediated apoptosis in vitro in primary CLL cells. In the study by Zhu et al. (2012) inhibition of Bcl-1 and Mcl-1 expression sensitized the CLL cells to ﬂudarabine-dependent cytotoxicity. Our study further confirms the results of the above reports and suggests that suppression of Bcl-1 and Mcl-1 expression by miRNA-15a can increase the sensitivity of CLL cells to ﬂudarabine.

Although miRNA-15a acts as a tumor suppressor gene through targeting critical molecules in CLL, few studies have directly linked miRNA-15a to chemoresistance of CLL. In CLL cases, down-regulated or absent of miRNA-15a resulted in increased levels of Bcl-2, Mcl-1, cyclin D1, cyclin D3 and CDK6 (Calin et al., 2008; Liu et al., 2008). In this study, transfection of miRNA-15a enhanced the sensitivity of CLL cells to fludarabine. So far, several studies have been conducted to investigate the role and relationship of miRNA with drug resistance. For example, Zhu et al. (2012) demonstrated that transfection of CLL cells with miRNA-34, miRNA-181a/b, miRNA-15a and miRNA-16-1 inhibits the expression of Mcl-1 and Bcl-2 and sensitize the CLL cells to ﬂudarabine-induced killing. Some other investigations of miRNA expressions in CLL patients showed that miRNA-221 and miRNA-181a were strongly up-regulated, and miRNA-29a was significantly down-regulated in fludarabine-resistant cases (Calin et al., 2005; Moussay et al., 2010). Likewise, Ferracin et al. (2010) assessed the changes in the expression of miRNAs in CLL patients prior to and following fludarabine therapy. The results of their study showed that miRNA-148a, miRNA-222 and miRNA-21 were significant up-regulated in non-responsive patients compared with patients sensitive to fludarabine. Moreover, a significant increase in caspase activity of human MEG-01 p53-mutant cell line was induced by anti-miRNA-21 and anti-miRNA-222 oligonucleotides, suggesting the pivotal role of these miRNAs in fludarabine resistance. However, our findings are in consistence with the above reports and show that miRNAs, especially miRNA-15a, can be useful in future therapeutic approaches by interfering with the drug resistance processes.

Our results showed that treatment with fludarabine induced cell apoptosis and increased caspase-3 activity in CLL-CII cells. Also, the suppression of Mcl-1 and Bcl-2 expression by miRNA-15a triggered apoptosis and enhanced the rate of fludarabine-induced apoptosis. The intrinsic or mitochondrial pathway of cell death is triggered by an array of stimuli such as cytotoxic drugs, radiation, oxidative stress and DNA damage. This pathway is regulated by the pro- and anti-apoptotic members of the Bcl-2 family proteins. In apoptotic conditions, the pro-apoptotic members such as Bak and Bax form a homodimer, leading to the change in the mitochondrial outer membrane permeability (MOMP), release of cytochrome c from mitochondria, and subsequently activation of caspases-3, -6 and -7. The anti-apoptotic proteins such as Bcl-2 and Mcl-1, by heterodimerising with Bak and Bax, inhibit apoptosis (Shahverdi et al., 2020a; Shahverdi et al., 2020b). Fludarabine induces apoptosis in vitro systems and in primary CLL cells by inhibiting several enzymes involved in DNA and RNA synthesis. It has been demonstrated that caspase-3 activation is induced by the fludarabine. However, the effect of fludarabine on the activation of the mitochondrial pathway of the apoptosis requires further exploration (Renatus et al., 2001; Danial, 2007). Moreover, it has been shown that over-expression of the anti-apoptotic proteins Bcl-1 and Mcl-1 is associated with resistance to ﬂudarabine in CLL patients (Robertson et al., 1996; Zhu et al., 2012). In confirmation of previous researches, we have shown that decreased expression of Bcl-1 and Mcl-1 by miRNA-15a enhanced the apoptotic effect of ﬂudarabine in the CLL cells. These findings suggest that miRNA-15a may increase fludarabine sensitivity through the internal pathway of apoptosis and caspase-3 activation.

In conclusion, the present study indicated that miRNA-15a acted in concert with fludarabine to exert synergistic anticancer efficacy against chronic lymphocytic leukemia, which attributed to the down-regulation of Mcl-1 and Bcl-2. In addition, our ﬁndings revealed that miRNA-15a could potentiate the execution of apoptosis induced by fludarabine. The underlying mechanism involved may be related to the intrinsic pathway of apoptosis and caspase activation. Collectively, the findings show that the combination of miRNA-15a and fludarabine can efficaciously induce the death of CLL cells, and may offer a promising therapeutic strategy for patients with CLL.

## Author Contribution Statement

Study concept and design: HK; Acquisition of data: NA, RA and MB; Analysis and interpretation of data: HK, MB and NM; Drafting of the manuscript: NA, RA and NM; Critical revision of the manuscript for important intellectual content: HK and MB; Funding recipients: HK
